# Antitumor activity of the selective cyclooxygenase-2 inhibitor, celecoxib, on breast cancer in *Vitro* and in *Vivo*

**DOI:** 10.1186/1475-2867-12-53

**Published:** 2012-12-19

**Authors:** Zhi-Jun Dai, Xiao-Bin Ma, Hua-Feng Kang, Jie Gao, Wei-Li Min, Hai-Tao Guan, Yan Diao, Wang-Feng Lu, Xi-Jing Wang

**Affiliations:** 1 Department of Oncology, the Second Affiliated Hospital, Medical School of Xi’an Jiaotong University, Xi’an, 710004, China

**Keywords:** Breast cancer, Cyclooxygenase-2, Anti-tumor, DMBA

## Abstract

**Background:**

Cyclooxygenase-2(COX-2) promotes carcinogenesis, tumor proliferation, angiogenesis, prevention of apoptosis, and immunosuppression. Meanwhile, COX-2 over-expression has been associated with tumor behavior and prognosis in several cancers. This study investigated the antitumor effects of the selective COX-2 inhibitor, Celecoxib, on breast cancer *in vitro* and *in vivo*.

**Methods:**

Human breast cancer MCF-7 and MDA-MB-231 cells were cultured with different concentration (10, 20, 40 μmol/L) of celecoxib after 0-96 hours *in vitro*. MTT assay was used to determine the growth inhibition of breast cancer cells in vitro. The expression of COX-2 on mRNA was measured by real-time quantitive PCR analysis. Flow cytometry was performed to analyze the cell cycle of MCF-7 cells. Levels of PGE2 were measured by ELISA method. The *in vivo* therapeutic effects of celecoxib were determined using rat breast cancer chemically induced by 7,12-dimethylben anthracene (DMBA).

**Results:**

The inhibition of proliferation of both MCF-7 and MDA-MB-231 cells in vitro by celecoxib was observerd in time and dose dependent manner. Celecoxib effectively down-regulated the expression of COX-2. The cell cycle was arrested at G0/G1, and rate of cells in S phase was obviously decreased. Levels of PGE2 were inhibited by Celecoxib. The tumor incidence rate of the celecoxib group was lower than that of the control group. In addition, the tumor latency period of the celecoxib group was longer than that of the control group.

**Conclusions:**

Celecoxib inhibited the proliferation of breast cancer cell lines *in vitro*, and prevented the occurrence of rat breast cancer chemically induced by DMBA. Therefore, celecoxib exhibits an antitumor activity and seems to be effective in anti-tumor therapy.

## Introduction

Cyclooxygenases(COX) exists in two isoforms, namely, COX-1 and COX-2. They are rate-limiting enzymes in the formation of prostaglandins from arachidonic acid. COX-1 is considered to be constitutively expressed, while COX-2 is highly inducible by various factors and is associated with tumorigenesis by enhancing angiogenesis [1,2], suppressing apoptosis [3], and promoting invasiveness as well as metastases [4].

COX-2 promotes carcinogenesis, tumor proliferation, angiogenesis, prevention of apoptosis, and immunosuppression [5]. COX-2 over-expression has been associated with tumor behavior and with prognosis in several cancers [6]. The selective inhibition of COX-2 activity in several animal models has been associated with a decrease of new blood vessel production in tumors, a decrease in new vessel formation, and an increase in tumor cell apoptosis [7]. Celecoxib is a paradigmatic selective inhibitor of COX-2. This anti-inflammatory drug has potent anti-tumor activity in a wide variety of human tumor types, such as colorectal, breast, and lung cancers [8-10].

The over-expression of COX-2 is associated with carcinogenesis, invasiveness, and with the metastasis of malignant tumors [11,12]. The roles of celecoxib in preventing and treating tumors have been attracting broad attention in recent years because of its selective and specific inhibition of COX-2 activity [13-16]. In this study, the inhibitory effect of celecoxib on the proliferation of the human breast carcinoma cell line MCF-7 was investigated *in vitro* and the breast cancer was chemically induced *in vivo*.

## Materials and methods

### Reagents

MCF-7 cell was purchased from the Shanghai Institute of Cell Biology at the Chinese Academy of Sciences (Shanghai, P.R.China). RPMI1640 medium (Gibco, USA); Fetal bovine serum(Gibco, USA); 7,12-dimethylbenanthracene (DMBA), Dimethyl sulfoxide (DMSO), Propidiumiodide(PI) and 3-(4,5- dimethylthiazol-2-yl) -2,5- diphenytetrazolium bromide (MTT) were purchased from Sigma Chemical (St. Louis, MO); Trizol (Invitrogen, USA);Celecoxib(Pfizer Pharmaceuticals Ltd, USA); Prostaglandin E2(PGE2) ELISA kit(Jingmei Biotech Co.,Ltd, China). Rats(Sprague–Dawley rats, female, age 45±5 days, weighting 110±10g) were purchased from the Experiment Animal Center, Medical School of Xi'an Jiaotong University, Xi’an, China (Animal Certificate Number: No.08-005 of Shanxi medical animal test centre).

### Cell culture and cell proliferation assay

Cells were cultured in RPMI-1640 medium(Gibco, USA) supplemented with 10% fetal bovine serum (Gibco, USA), 1 ×10^5^ U/L penicillin and 100 mg/L streptomycin in a humidified atmosphere with 5% CO_2_ incubator at 37°C. The cells were subcultured until reaching logarithmic growth phase.

MTT assay was used to determine the effect of Celecoxib on the proliferation of MCF-7 cells. MCF-7 cells were seeded at a concentration of 5×10^3^ cell /well in 96-well plate, and grown at 37°C, 5% CO_2_ incubator until adherence. After an overnight incubation in starvation medium containing 0.5% FBS, the cells on the culture plate were divided into groups on the basis of parallel lines, each group had four wells in one line for each group. At the end of the treatment, 20 μl MTT (5 mg/ml) was added and the cells were incubated for another 4 hours. 200 μl of DMSO was added to each well after removing the supernatant. After shaking the plate for 10 mins. in the shaking board, cell viability was obtained by measuring the absorbance at 490 nm wavelength using Enzyme-labeling instrument (Bio-Tek ELX800, USA), this assay was done triplicate. The inhibition rate was calculated using the following formula [17]:

Inhibition rate(%) =[1-(average absorbance of experimental group/average absorbance of blank control group)] × 100%.

### Real-time quantitative RT-polymerase chain reaction assay for COX-2 expression

MCF-7 cells were seeded in 6-well plates and treated with concentration gradient Celecoxib (0, 10,20,40 μmol/L) separately for 0-96 h. As previously described [18], cells collected at specified time were used to extract total RNA using the Trizol reagent following the manufacturer’s instructions. RNA was reverse-transcribed into cDNA using a Primescript™ RT reagent kit according to the manufacturer’s instructions. Real-time quantitative polymerase chain reaction (PCR) was carried out with the SYBR Green fluorescent dye method, and a Rotor Gene 3000 real-time PCR apparatus. COX-2 primer sequence (Invitrogen CO): 5^′^- ATCCTTGCTGTTCCCACCA-3^′^ (sense) and 5^′^-CTTTGACACCCAAGGGAGT-3^′^ (anti-sense). β-actin, its primer sequence was 5^′^-GTTGCGTTACACCCTTTCTTG-3^′^(sense), 5^′^-TGCTGTCACCTTCACC GTTC-3^′^ (anti-sense). β-actin was used as an internal control to evaluate the relative expressions of COX-2. The PCR conditions were as follows: a pre-denaturing at 95°C for 2 min, followed by 45 cycles of denaturation at 95°C for 10 s, annealing/extension at 60°C for 20 s. The amplification specificity was checked by melting curve analysis. The PCR products were visualized by gel electrophoresis to confirm the presence of a single product with a correct size. The 2-^ΔΔCT^ method was used to calculate the relative abundance of target gene expression generated by Rotor-Gene Real-Time Analysis Software 6.1.81. For each cDNA, the target gene mRNA level was normalized to β -actin mRNA level. The experiments were performed for three times.

### Determination of PGE2 synthesis

As previously described [19], MCF-7 Cells were grown in 12-well plates overnight. 30 min before harvesting of culture media, the culture media of the cells were changed to new media, and then these culture media were centrifuged to remove cell debris. Cell-free culture media were collected at indicated times and PGE2 levels were determined by competitive enzyme-linked immunosorbent assay (ELISA) as described by the kit manufacturer (Cayman Chemical, Ann Arbor, MI, USA) using an ELISA reader (μQuant; Biotek Instruments, Inc, Winooski, VT, USA).

### Cell cycle analysis by FCM

MCF-7 cells were incubated at 5 × 105 cells/well in 6-well plates, treated with a homologous drug for 48 h. The detached and attached cells were harvested and fxed in 70% ice-cold ethanol at -20°C overnight. After fixation, cells were washed with PBS, resuspended in 1 mL PBS containing 1 mg/mL RNase (Sigma) and 50 μg/mL PI (Sigma), and incubated at 37°C for 30 min in the dark. Samples of 10 000 cells were then analyzed for DNA content by FACScan flow cytometry (Beckman, USA), and cell cycle phase distributions were analyzed with the CellQuest acquisition software(BD Biosciences).

### Antitumor activity in vivo

All the animals were maintained under standard environmental conditions and provided with food and water ad libitum. All the animals were fed with a normal pellet diet one week prior to the experimentation.

Female Sprague–Dawley rats were gavaged with 60 mg of DMBA/kg body weight, which was sufficient to cause 100% tumor incidence in the rats over the course of the study, as described by Whitsett et al. [20]. The DMBA was dissolved in olive oil at a stock solution of 30 mg/ml.

After modeling, 90 rats were randomly divided into three groups, namely, the control group(normal diet after modeling), which was considered as the negative control, the positive control (tamoxifen) group (4 mg/kg of tamoxifen was put in drinking water), and celecoxib group(solved in the oleum maydis, 1000 mg/kg of celecoxib was put in forage). The breasts of the rats were assessed twice a week, when palpable breast neoplasm appeared. The number of breast neoplasms and their changes in size were recorded.

After 120 days, the experiment was stopped. A vaginal smear examination was performed to confirm that the rats were in the dioestrus period. The rats were anesthetized by an intraperitoneal injection of 5% urethane. Then, every mammary gland with surrounding skin and hypodermia were dissected.The mammary glands were cut half open to observe the tumor shape and to count the number of tumors. The size of the tumors was measured using a 1mm precision sliding caliper. The tumor incidence rate of each group was calculated. The tumor volumes was calculated according to the following equation: length × weight × height × π/6 [21]. If one rat had many tumors, the volume of this rat was the sum of all its tumors.

The specimens were fixed in 10% neutral formalin, embedded in paraffin and stained with hematoxylin-eosin for the histological examination. The diagnosis of rat breast cancer was performed according to the diagnostic code of experimental rat breast cancer [22].

### Statistical analysis

All values were expressed as the mean ± standard deviations (SD). Statistical analysis was performed with one-way analysis of variance (ANOVA) and student *t* test using the statistical software SPSS 13.0. *P*<0.05 was considered as statistically significant.

## Results and discussions

### Celecoxib inhibited the proliferation of breast cancer cells in vitro

**Figure 1:**
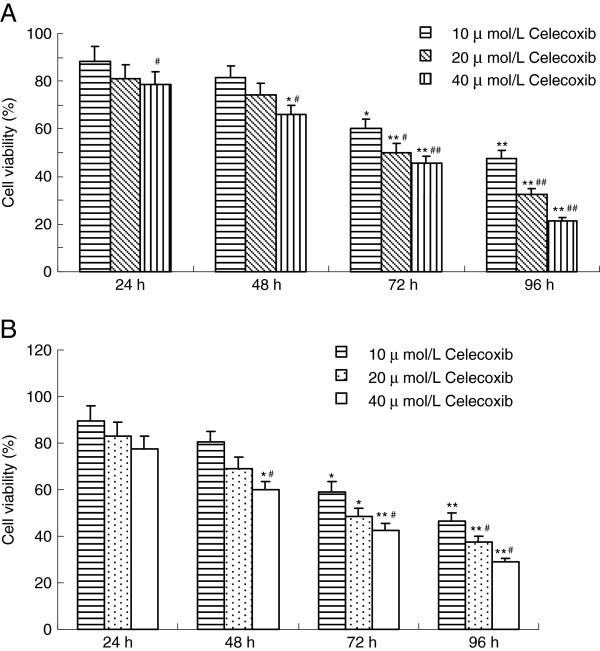
**Growth inhibiting effects of Celecoxib in MCF-7 cells (A) and MDA-MB-231 cells (B). **Cell viability was determined by MTT method. This assay was performed in triplicate. Dose- and time-dependent inhibition of cell growth could be observed after 96 h (*P*<0.05, ANOVA analysis). *P<0.05, ** *P*<0.01 *vs*. 24 h; ^#^*P*<0.05, ^##^*P*<0.01 *vs*.10 μmol/L celecoxib.

The anti-proliferative effect of celecoxib on two breast cancer cell lines, MCF-7 cells (ER –positive) and MDA-MB-231 (ER-negative), were examined using MTT assays. Cells were treated with medium and different doses of celecoxib, and the inhibition rate was evaluated after 0 h to 96 h. Celecoxib in the high dose and medium dose groups could significantly inhibit the proliferation of both MCF-7 cells and MDA-MB-231 cells. As shown in Figure 1, the inhibitory rates of celecoxib on cell growth were (62.6±4.5)%, (67.5±4.8)%, (78.7±6.3)% in MCF-7 cells, and 53.5±3.7)%, (62.3±4.5)%, (70.9±7.1)% in MDA-MB-231 cells, when the cells were treated with different doses of celecoxib for 96 hours. Thus, Celecoxib inhibited breast cancer cell proliferation in a dose- and time-dependent manner.

Celecoxib is known to induce apoptosis [23], but its effect on proliferative is not conclusive. For example, some researches reported that celecoxib did not affect tumor cell proliferation in primary adenocarcinomas and ductal carcinoma in situ of the breast in vivo [24,25]. However, celecoxib could prevent the development of breast cancer with ER-negative and HER-2-positive status [26]. These results indicate that the effect on proliferative of celecoxib is related with tumor`s molecular phenotype possibly. In this study, we choose a ER –positive breast cancer cell line (MCF-7) and a ER –negative breast cancer cell line (MDA-MB-231), and found celecoxib could significantly inhibit the proliferation of both MCF-7 cells and MDA-MB-231 cells in a dose- and time- dependent manner.

### COX-2 mRNA expression detected by real-time qPCR

**Figure 2:**
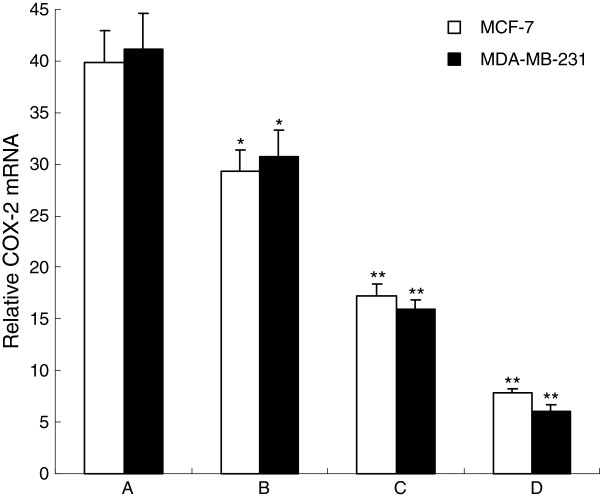
**Effect of Celecoxib on expression of COX-2 mRNA of MCF-7 cells by real-time qPCR analysis. A**: Control group; **B**: 10 μmol/L Celecoxib; **C**: 20 μmol/L Celecoxib; **D**: 40 μmol/L Celecoxib. The expression of COX-2 mRNA in MCF-7 cells were determined by real-time qPCR after treated with 0-40μmol/L celecoxib for 48 h. This assay was done triplicate. Values represent means ± standard deviations and were determined using the Student’s *t*-test. *P<0.05,** *P*<0.01 *vs.* control.

Real-time qPCR assay was used for COX-2 mRNA expression. It revealed that COX-2 mRNA was highly expressed in normal breast cancer cells. What’s more, as celecoxib concentration increased, the mRNA expression of COX-2 gradually decreased. As shown in Figure 2, the amount of COX-2 mRNA in both MCF-7 cells and MDA-MB-231 cells after celecoxib treatment were significantly decreased in a dose-dependent manner.

It was previously reported that COX-2 is expressed in most human cancers including those of the breast, and administration of selective COX-2 inhibitors in humans may reduce the risk of cancer development [27]. Our date indicated that cell viability was gradually declined as celecoxib concentration increased. As can been seen, the lowest COX-2 mRNA was in 40 μmol/L group. Therefore, celecoxib could suppress expression of COX-2 to inhibit cell proliferation. This results points out the efficacy of celecoxib against breast cancer growth.

### Effects of celecoxib on the PGE2 level of MCF-7 cells by ELISA

Prostaglandin E2 (PGE2) is an important mediator in tumor-promoting inflammation [28]. The major mechanism of COX-2 in stimulating tumorigenesis is its product, PGE2. PGE2 promotes tumor cell proliferation, induces VEGF up-regulating, and inhibits tumor cell apoptosis as well as immune function [29]. Tari et al. [30] reported that COX-2 induced PGE2 to stimulate the activities of protein kinases A and C and induced tamoxifen resistance in ER alpha-positive breast cancer cells selectively. However, the COX-2 selective inhibitor celecoxib can inhibit tumorigenesis and tumor development through these ways.

**Figure 3:**
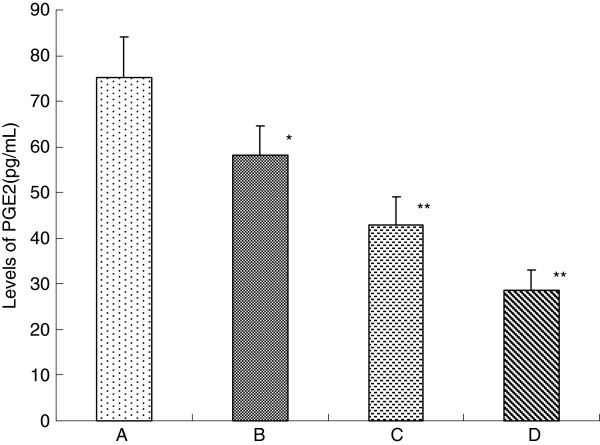
**Effects of celecoxib on the PGE2 level of MCF-7 cells by ELISA. A**: control group; **B**: 10 μmol/L celecoxib; **C**: 20 μmol/L celecoxib; **D**: 40 μmol/L celecoxib. PGE2 levels of MCF-7 cells were determined by ELISA after treated with 0-40 μmol/L celecoxib for 48 h. This assay was done triplicate. Values represent means ± standard deviations and were determined using the Student’s *t*-test. *P<0.05, ** *P*<0.01 *vs*. control.

In this study, the PGE2 level of MCF-7 cells was determined with ELISA analysis. As shown in Figure 3, the PGE2 level of MCF-7 cells in the control group and in the 10 μmol/L to 40 μmol/L celecoxib groups were (75.32±8.73), (58.15±6.56), (42.84±6.12) and (28.65±4.33) pg/mL, respectively. PGE2 levels in the celecoxib therapy groups were significantly lower than that in the control group. Furthermore, the PGE2 level gradually decreased in a dose-dependent manner (*P* <0.01).

### Effects of celecoxib on the cell cycle distribution by flow cytometry

Celecoxib may exert an inhibitory effect on the enhanced radiation-induced G2/M arrest in the COX-2-overexpressing cells. This effect may allow the arrested cells to enter mitosis and die after radiation [31]. It has been found that a low dose of celecoxib (5 μM to 10 μM) could induce G2/M arrest, followed by the induction of apoptosis in the transformed cells but not in the normal cells. Growth inhibition was related to the COX-2 function with 90% to 95% reduction in PGE2 production [32]. However, Liu et al. [33] holds a different opinion that celecoxib can induce apoptosis and cell-cycle arrest at the G0/G1 checkpoint in the nasopharyngeal carcinoma cell lines, which is associated with a significantly reduced STAT3 phosphorylation.

**Figure 4:**
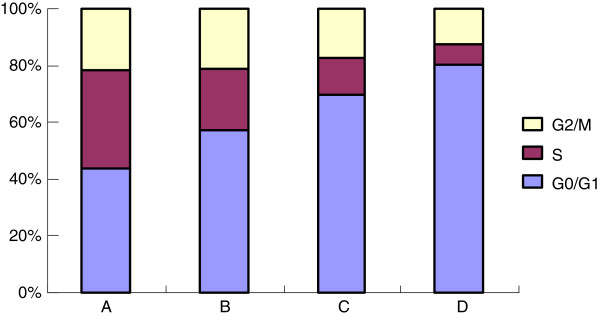
**Effects of celecoxib on the cell cycle of MCF-7 cells by flow cytometry. A**: control group; **B**: 10 μmol/L celecoxib; **C**: 20 μmol/L celecoxib; **D**: 40 μmol/L celecoxib. The cell cycle distributions in MCF-7 cells were determined by PI staining and flow cytometric analysis after treated with 0-40 μmol/L celecoxib for 48 h. Results presented were representative of three independent experiments.

In the present study, the effects of celecoxib on cell cycles were analyzed using flow cytometry. The percentage of cells in the celecoxib therapy groups significantly decreased at the S phase and increased at the G0/G1 phase. These results suggest that celecoxib can induce cell cycle arrest at the G0/G1 phase in MCF-7 cells (Figure 4). Besides, the cells at the G2/M phase significantly decreased in the 40 μmol/L celecoxib group compared with that in the control group.

### Anti-tumor effects of celecoxib on DMBA-induced breast cancer

Celecoxib has a striking chemopreventive activity. It can inhibit preneoplastic lesions during hepatocarcinogenesis *in vivo*, which suggests that celecoxib effects are mediated by PGE2-independent mechanisms [34]. A low dose of celecoxib can augment CDDP-induced growth inhibition of Tca8113 cells and its xenograft in Balb/c nude mice [35]. It is reported by Nakatsug et al. [21] that 400 ppm of nimesulide could degrade tumor incidence rate, volume and multisitus rate. This study found that celecoxib could inhibit rat carcinogenesis and cancer development. With a dosage of 1000 ppm, celecoxib decreased the incidence rate, average tumor number and tumor volume with statistical significance (*P*<0.05), compared with that of tumor control group. As *in vitro*, celecoxib inhibited MCF-7 cell proliferation in a dose- and time-dependent manner, while *in vivo*, celecoxib could inhibit rat carcinogenesis and cancer development in a dose-dependent manner. Abou Issa et al. [9] observed the preventive effect of celecoxib on 7,12-dimethylben anthracene (DMBA)-induced rat breast cancer. When treated with 250 ppm, 500 ppm, 1000 ppm and 1500 ppm celecoxib, the incidence rate was 80%, 50%, 45% and 25% respectively, compared with that of 100% incidence rate in control group (*P*<0.001).

**Figure 5:**
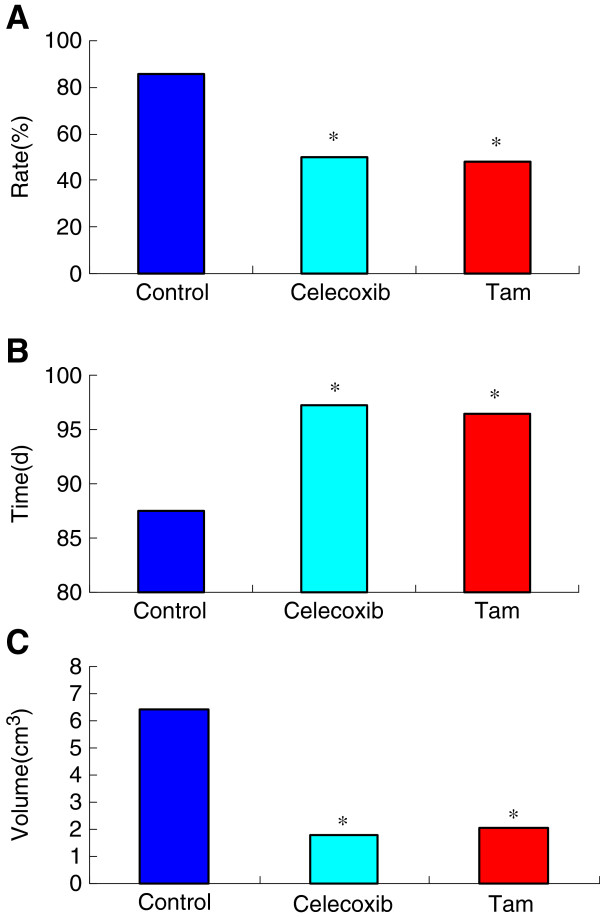
**Anti-tumor effects of celecoxib on DMBA induced breast cancer. A**. the incidence rate of the different groups; **B**. the tumor latency period of the different groups; **C**. the average tumor volumes of the different groups. Values represent means ± standard deviations and were determined using the Student’s *t*-test. ^*^P < 0.05 *vs* tumor control group.

In the present study, celecoxib was found to be capable of inhibiting rat carcinogenesis and cancer development. The tumor incidence rate of each group was 85.71% (24/28) in the control group, 50.00% (14/28) in the celecoxib group, and 48.15% (13/27) in the tamoxifen group. As shown in Figure 5, the tumor latency period of the celecoxib group was significantly longer than that of the control group (*P* < 0.05). In addition, no significant difference was found between the celecoxib and tamoxifen groups (*P* > 0.05).

The average tumor numbers of each group were as follows: 3.50±1.62(1-7) pieces in the control group; 1.77±0.73(1-3)pieces in the tamoxifen group; and 1.71±0.61(1-2) pieces in the celecoxib group. The average tumor numbers of the celecoxib and tamoxifen groups were less than that of the control group (*P* < 0.05). No significant difference was found between the two treatment groups (*P* > 0.05).

The average tumor volumes of each group were 6.42±3.96cm^3^ in the control group, 1.78±0.71cm^3^ in the tamoxifen group, and 2.05±1.04cm^3^ in the celecoxib group. No significant difference was found between the tumor volumes of the celecoxib and tamoxifen groups (*P* > 0.05). The tumor volumes of the two treatment groups decreased significantly compared with the tumor control groups (*P* < 0.05).

### Histopathological observation

**Figure 6:**
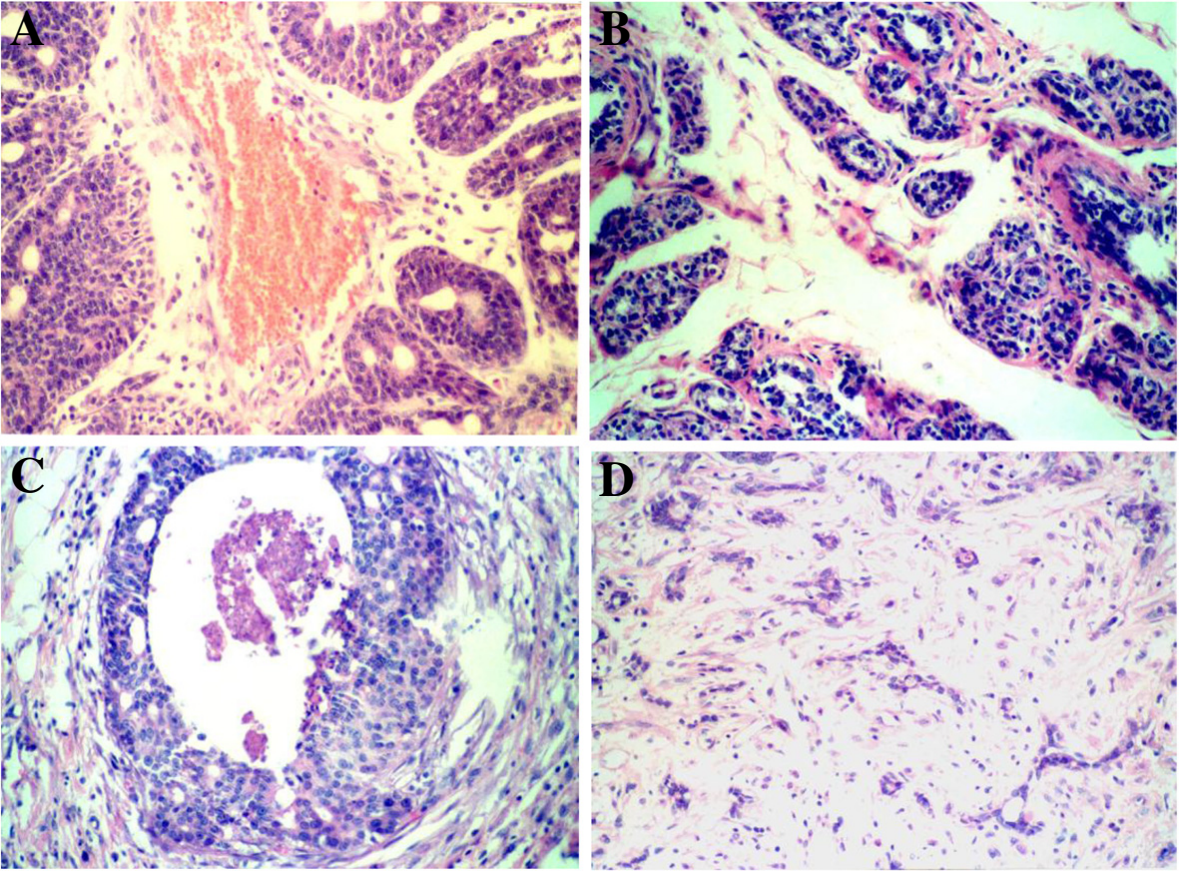
**Breast sections of the different groups. A**. Infiltrating ductal cancer in control group; **B**. Atypical hyperplasia in control group; **C**. Infiltrating ductal cancer in celecoxib group; **D**. Hyperplasia in celecoxib group (HE×200).

The tissues of the control group presented an infiltrating ductal carcinoma, showing cancer nest, obvious nuclear atypia and nuclear division. A few gland-like structures and stroma were also observed (Figure 6A). The medullary carcinoma was composed of cancer cells and had ductless glands. The specimens without tumorigenesis showed different degrees of lobuli mammae hyperplasia and glandular epithelium atypical hyperplasia. The slight degree of hyperplasia showed an expanded or increased intralobulus and interlobulus fibrous tissue and gland alveolus. However, acinous cells were still in the monolayer. The atypical hyperplasia showed acinous cells that were arranged disorderly and in a multilayer, an increased karyoplasmic ratio, and changed nuclear atypia (Figure 6B).

In the experimental groups, the infitrating ductal carcinoma had more gland-like structures and stroma wherein the cells were dispersed. A few nuclear atypia and nuclear division were also observed (Figure 6C). The specimens that did not cancerate showed no or a slight degree of glandular epithelium hyperplasia, increased intralobulus and interlobulus fibrous tissue, and lesser gland alveolus and ducts (Figure 6D).

## Conclusions

In conclusion, celecoxib inhibited the proliferation of breast cancer cell lines *in vitro*. Furthermore, the inhibitory effect of celecoxib on the proliferation of breast cancer cells *in vitro* was observed in a dose- and time-dependent manner. The cell cycle was arrested at G0/G1, and the rate of cells in the S phase was obviously decreased. In addition, celecoxib could prevent the occurrence of rat breast cancer *in vivo*. Therefore, celecoxib exhibits an antitumor activity and seems to be effective in anti-tumor therapy. However, further studies are needed to clarify the detailed mechanism involved in the antitumor effects of celecoxib.

## Competing interest

The authors declare that there are no conflicts of interest in relation to this article.

## Authors’ contributions

DZJ and WXJ designed the research. DZJ, MXB, GJ, MWL and DY performed the experiments throughout this research. LXX, KHF and GHT contributed to the reagents, and participated in its design and coordination. DZJ and GJ analyzed the data; DZJ and MXB wrote the paper. Co-first authors: DZJ and MXB. All authors have read and approved the final manuscript.
